# Identification of KCC2 Mutations in Human Epilepsy Suggests Strategies for Therapeutic Transporter Modulation

**DOI:** 10.3389/fncel.2019.00515

**Published:** 2019-11-15

**Authors:** Phan Q. Duy, Wyatt B. David, Kristopher T. Kahle

**Affiliations:** ^1^Department of Neurosurgery, Yale University School of Medicine, New Haven, CT, United States; ^2^Medical Scientist Training Program, Yale University School of Medicine, New Haven, CT, United States; ^3^Department of Genetics, Yale University School of Medicine, New Haven, CT, United States; ^4^Departments of Pediatrics and Cellular & Molecular Physiology, Yale University School of Medicine, New Haven, CT, United States; ^5^Yale-Rockefeller NIH Centers for Mendelian Genomics, Yale University, New Haven, CT, United States; ^6^Yale Stem Cell Center, Yale School of Medicine, New Haven, CT, United States

**Keywords:** KCC2, SLC125A, epilepsy, seizure, neuronal excitability, neurodevelopment

## Abstract

Epilepsy is a common neurological disorder characterized by recurrent and unprovoked seizures thought to arise from impaired balance between neuronal excitation and inhibition. Our understanding of the neurophysiological mechanisms that render the brain epileptogenic remains incomplete, reflected by the lack of satisfactory treatments that can effectively prevent epileptic seizures without significant drug-related adverse effects. Type 2 K^+^-Cl^−^ cotransporter (KCC2), encoded by *SLC12A5*, is important for chloride homeostasis and neuronal excitability. KCC2 dysfunction attenuates Cl^−^ extrusion and impairs GABAergic inhibition, and can lead to neuronal hyperexcitability. Converging lines of evidence from human genetics have secured the link between KCC2 dysfunction and the development of epilepsy. Here, we review KCC2 mutations in human epilepsy and discuss potential therapeutic strategies based on the functional impact of these mutations. We suggest that a strategy of augmenting KCC2 activity by antagonizing its critical inhibitory phosphorylation sites may be a particularly efficacious method of facilitating Cl^−^ extrusion and restoring GABA inhibition to treat medication-refractory epilepsy and other seizure disorders.

## Introduction

A seizure is a transient increase in the brain’s electrical activity that may be triggered by a variety of factors, including medications (Chen et al., 2016), metabolic alterations (Imad et al., 2015), and infections (Zoons et al., 2008). When seizures arise spontaneously, they are considered to be epileptic. Epilepsy is the most common serious brain disorder worldwide (World Health Organization, 2019) characterized by recurrent and unprovoked seizures that can cause loss of consciousness and/or abnormal motor behavior depending on the afflicted brain region (Stafstrom and Carmant, 2015). The disorder affects 0.5% of the general population (Sander and Shorvon, 1996) and is associated with increased rates of mortality (Zieliński, 1974), cognitive impairment (Aldenkamp, 2006), and psychosocial dysfunction (Pershad and Siddiqui, 1992) at an annual cost to the US economy of $12 billion (Begley et al., 2000).

Epilepsy is classically thought to arise from an imbalance between neuronal excitation and inhibition, leading to a hyperexcitable state that is prone to seizure activity. Antiepileptic drugs (AEDs) are the mainstay therapy for epilepsy that aim to restore this balance in neuronal excitability by either suppressing excitatory neurotransmission or augmenting inhibition. Despite decades of medical research and development of novel third-generation AEDs, a third to a half of epilepsy patients on medications continue to have seizures (medication refractory epilepsy; Kwan and Brodie, 2000; Shorvon and Luciano, 2007; Cascino, 2008). Furthermore, AEDs often exert significant drug-related adverse effects, including dizziness, nausea, fatigue, depression, learning and memory impairments, and ataxia (Perucca and Meador, 2005). The lack of a truly satisfactory AED reflects our incomplete understanding of epileptogenesis, the set of pathogenic alterations that render neuronal networks hyperexcitable and thus vulnerable to pathological seizure activity. There is an urgent clinical need for novel insights into cellular and molecular mechanisms of epileptogenesis in order to develop more efficacious AEDs that can achieve seizure freedom with minimal or no side effects.

Human genetic studies have associated mutations in the neuron-specific type 2 K^+^/Cl^−^ cotransporter KCC2 with the development of epilepsy (Kahle et al., 2014; Puskarjov et al., 2014; Stödberg et al., 2015; Saitsu et al., 2016; Saito et al., 2017; Till et al., 2019). Preclinical studies suggest that modulation of KCC2 activity by targeting critical regulatory domains may be exploited to suppress seizure activity (Moore et al., 2018), highlighting the key role of KCC2 in the regulation of neuronal excitability in physiological and epileptogenic states. In this article, we review the KCC2 mutations that are associated with the development of epilepsy in humans. We also discuss the therapeutic ramifications of these findings and postulate that KCC2 may be a potentially powerful therapeutic target for the development of novel AEDs to treat refractory epilepsy.

## Type 2 K^+^-Cl^−^ Cotransporter (KCC2) in Chloride Homeostasis and Synaptic Inhibition

Neuronal excitability describes the propensity of a postsynaptic neuron to generate an action potential, a rapid rise and fall in membrane potential that occurs when the neuron reaches a threshold level of membrane depolarization. Consequently, neuronal excitability is governed by a dynamic balance between excitatory and inhibitory inputs. Excitatory inputs are depolarizing and thus raise the neuronal membrane potential towards the threshold, whereas inhibitory inputs are hyperpolarizing and lower the potential away from threshold. In the central nervous system, neuronal inhibition occurs primarily *via* activation of ligand-gated γ-aminobutyric acid (GABA) type A receptors (GABA_A_Rs) that are highly permeable to Cl^−^, and to a lesser extent, HCO_3_^−^ (Kaila and Voipio, 1987). Ligand binding to GABA_A_Rs on the postsynaptic neuron opens a central pore to trigger a hyperpolarizing Cl^−^ influx that lowers the probability of action potential being generated by neuron. The strength of synaptic inhibition is thus dependent on a low intra-neuronal concentration of Cl^−^, which provides the basis for an electrochemical gradient that permits passive movement of Cl^−^ through the plasma membrane upon GABA_A_R activation.

The electroneutral K^+^/Cl^−^ cotransporter KCC2 (encoded by *SLC12A5*) is a key determinant of Cl^−^ homeostasis in neurons of the central nervous system (Kahle and Delpire, 2016; Moore et al., 2017). Under normal physiological settings, KCC2 uses the outwardly directed K^+^ gradient generated by the N^+^-K^+^ ATPase pump to extrude Cl^−^ against its electrochemical gradient from neuronal cells in humans and thus maintains low intra-neuronal Cl^−^ concentrations required for hyperpolarizing GABAergic currents. In a likely over-simplified but useful scheme, intra-neuronal Cl^−^ concentrations are high during early brain development secondary to low KCC2 activity (Li et al., 2002; Stein et al., 2004) and high influx of Cl^−^ *via* Na^+^-K^+^-Cl^−^ cotransporter 1 (NKCC1; Plotkin et al., 1997; Yamada et al., 2004) in young neurons, leading to membrane depolarization following ligand binding to GABA_A_R (Ben-Ari et al., 1989). As the brain matures, NKCC1 activity is downregulated whereas KCC2 activity is upregulated (Plotkin et al., 1997; Stein et al., 2004), leading to hyperpolarizing GABAergic responses, though recent data shows that the expression changes of these molecules vary within the heterogeneous neuronal populations within the brain (Sedmak et al., 2016). Although other KCC isoforms exist, KCC2 is unique in that its expression is primarily localized to central nervous system neurons (Williams et al., 1999; Payne et al., 2003) and it remains constitutively active even under isotonic conditions (Khirug et al., 2005; Mercado et al., 2006). Importantly, at least in the setting of normal neurophysiology, KCC2 is able to remove extra Cl^−^ introduced by GABAergic neurotransmission and thus recover low intracellular Cl^−^ levels in neurons (Kaila et al., 2014; Doyon et al., 2016). These properties indicate that KCC2 is a major extruder of Cl^−^ in mature neurons that establishes the inwardly directed Cl^−^ electrochemical gradient across the plasma membrane necessary for the emergence and maintenance of inhibitory hyperpolarizing responses upon activation of GABA_A_Rs.

## KCC2 Mutations and Human Epilepsy

The importance of KCC2 in maintaining the strength of synaptic inhibition highlights its potential involvement in epilepsy, a disorder of neuronal hyperexcitability that has been thought to arise from failed neuronal inhibition. Preclinical studies in multiple organisms show that genetic KCC2 deficiency results in diminished Cl^−^ extrusion, neuronal hyperexcitability, and epileptic seizures (Hübner et al., 2001; Hekmat-Scafe et al., 2006; Tanis et al., 2009). Accordingly, downregulation of KCC2 levels is observed in human idiopathic epilepsy (Huberfeld et al., 2007). Recent studies have now demonstrated the presence of KCC2 mutations in human epilepsy patients, providing strong evidence for the role of KCC2 in seizure disorders. All of the KCC2 mutations discovered in human epilepsy thus far are summarized in Table 1.

**Table 1:** KCC2 (SLC12A5) mutations in human epilepsy.

Exon	NT change	AA change	Type	Inheritance	Phenotype	Ethnicity	Reference
21	c.2855G>A	p.R952H	Missense	AD	IGE; Febrile Seizures	French Canadian; Australian	Kahle et al. (2014) and Puskarjov et al. (2014)
23	c.3145C>T	p.R1049C	Missense	AD	IGE	French Canadian	Kahle et al. (2014)
9	c.1277T>C	p.L426P	Missense	AR, compound heterozygous	EIMFS	Swedish	Stödberg et al. (2015)
13	c.1625G>A	p.G551D	Missense			
8	c.932T>A	p.L331H	Missense	AR, homozygous	EIMFS	Pakistani	Stödberg et al. (2015)
3	c.279 + 1G>C	p.E50_Q93del	Deletion	AR, compound heterozygous	EIMFS	Japanese	Saitsu et al. (2016)
6	c.572C>T	p.A191V	Missense			
8	c.967T>C	p.S323P	Missense	AR, compound heterozygous	EIMFS	Malaysian	Saitsu et al. (2016)
10	c.1243A>G	p.M415V	Missense			
8	c.953G>C	p.W318S	Deletion	AR, compound heterozygous	EIMFS	Japanese	Saitsu et al. (2016)
18	c.2242_2244del	p.S748del	Missense			
9	c.1196C>T	p.S399L	Missense	AR, compound heterozygous	EIMFS	Japanese	Saito et al. (2017)
20	c.2639G>T	p.R880L	Missense			
11	c.1417G>A	p.V473I	Missense	AD	IGE	Hungarian	Till et al. (2019)

*AA, amino acid; AD, autosomal dominant; AR, autosomal recessive; EIMFS, epilepsy of infancy with migrating focal seizures; IGE, idiopathic generalized epilepsy; NT, nucleotide*.

### Idiopathic Generalized Epilepsy 14 (OMIM# 616685, Autosomal Dominant)

Kahle et al. (2014) used a targeted DNA-sequencing approach to screen the cytoplasmic C-terminal region of SLC12A5 which is an important regulatory region of transporter function. They identified two different heterozygous missense variants in *SLC12A5* (R952H, 606726.0004 and R1049C, 606726.0005) that were enriched among individuals of French Canadian origin with idiopathic generalized epilepsy-14 (EIG14; 616685) compared to controls. Both variants exhibited reduced Cl^−^ extrusion capacity, although unlike the R952H variant, the R1049C variant exhibited normal surface expression with decreased intrinsic cotransporter activity. Both variants also showed decreased phosphorylation of the serine 940 (S940) residue (Kahle et al., 2014), which normally promotes KCC2 activity (Lee et al., 2011). The overall effect impaired the function of KCC2. The variants were inherited from an unaffected parent in several cases, consistent with incomplete penetrance, consistent with other large genomic studies of human idiopathic generalized epilepsy (Mefford et al., 2011). Puskarjov et al. (2014) reported the R952H mutation in an Australian family with early childhood onset of febrile seizures. Segregation of the variant in this kindred was difficult because of uncertain phenotyping, but there was some evidence of incomplete penetrance. Electrophysiological and biochemical assays suggest that the R952H variant exhibits impaired Cl^−^ extrusion likely due to reduced surface expression. Overexpression of this variant in KCC2-deficient mouse cortical neurons failed to rescue defects in dendritic spine development, suggesting a potential role of the R952H variant in formation and maturation of cortical dendritic spines. Puskarjov et al. (2014) suggested that the decrease in KCC2-dependent hyperpolarizing inhibition would promote seizures, and that decreased dendritic spine formation could lead to desynchronization of overall excitability. Importantly, the function of KCC2 in the dendritic spine does not depend on transporter function but rather involves interactions between KCC2 and other proteins (Llano et al., 2015). The most recent study has identified a new missense KCC2 variant, V473I, that causes IGE in a Hungarian patient who is heterozygous for the mutation (Till et al., 2019).

### Early Infantile Epileptic Encephalopathy 34 (OMIM# 616645, Autosomal Recessive)

The strongest genetic evidence for KCC2 dysfunction in epilepsy is demonstrated by studies of patients in families with a severe infantile epilepsy syndrome termed epilepsy of infancy with migrating focal seizures (EIMFS; Stödberg et al., 2015; Saitsu et al., 2016; Saito et al., 2017). To date, nine probands with monogenetic KCC2-related EIMFS have been reported. By whole-exome sequencing of two unrelated families, Stödberg et al. (2015) discovered that affected children with EIMFS harbored biallelic *SLC12A5* loss-of-function mutations. Two affected children from a consanguineous family harbored the homozygous mutation L311H that localizes to an extracellular loop. The L426P (localizing to a transmembrane domain) and G551D (localizing to an intracellular loop) variants were found as compound heterozygous mutations in two affected children from another family. All of the KCC2 mutations identified by Stödberg et al. (2015) resulted in diminished Cl^−^ extrusion and reduced cell surface expression. Follow-up studies identified eight additional recessive KCC2 mutations that cause EIMFS (Saitsu et al., 2016; Saito et al., 2017), including the E50_Q93del variant that causes skipping of exon 3 and the S749del variant that causes an amino acid deletion (Saitsu et al., 2016). Functional characterization of some of these EIFM-causing KCC2 mutations (E50_Q93del, A191V, S323P, M415V) suggested that they attenuated neuronal Cl^−^ extrusion without altering cell surface expression and distribution (Saitsu et al., 2016). It is important to note that all of the homozygous or compound heterozygous EIMFS patients inherited KCC2 mutations from unaffected heterozygous parents, in contrast to the R952H and R1049 variants that were sufficient to cause epilepsy disorders in heterozygous individuals (Kahle et al., 2014; Puskarjov et al., 2014). The reasons underlying these phenotypic differences are not understood, although some have hypothesized that mutations located in different KCC2 domains exert different effects on functional activity that underlie variations in phenotypic manifestations (Kahle et al., 2016a).

## Augmenting KCC2 Activity to Restore Synaptic Inhibition as A Therapeutic Avenue for Epilepsy

The presence of KCC2 mutations in human epilepsy indicates that accumulation of intracellular Cl^−^ secondary to KCC2 dysfunction may be responsible for driving neuronal hyperexcitability underlying the development of epilepsy syndromes. The association between loss-of-function KCC2 mutations and epilepsy also suggests that augmenting KCC2 activity to enhance Cl^−^ extrusion may confer the opposite effect of rendering neuronal cells more resistant to seizures, representing a potentially powerful therapeutic avenue for idiopathic epilepsy. Indeed, high levels of neural activity due to seizures may promote the intracellular accumulation of Cl^−^ that exceeds the normal Cl^−^ extrusion capacity of KCC2, leading to GABAergic depolarizing currents and loss of synaptic inhibition that underlie epileptogenesis (Ellender et al., 2014; Magloire et al., 2019). Augmenting KCC2 function could theoretically extrude excessive Cl^−^ and restore neuronal inhibition in hyperexcitable states.

Targeting critical phosphorylation sites of KCC2 regulation is one promising strategy to enhance KCC2 function for therapeutic benefit. KCC2 activity is bidirectionally regulated at key phosphorylation sites: S940 phosphorylation increases KCC2 function (Lee et al., 2011), whereas phosphorylation of T906 and T1007 inhibits its function (Rinehart et al., 2009). Dephosphorylation of T906 and T1007 upregulates Cl^−^ extrusion from neurons (Friedel et al., 2015; Titz et al., 2015; Heubl et al., 2017). To test the hypothesis that increasing KCC2 activity is anticonvulsant *in vivo*, Moore et al. (2018) generated knock-in mice in which threonines 906 and 1007 were substituted for alanines (KCC2-T906A/T1007A) to genetically prevent phospho-dependent inactivation, resulting in higher basal neuronal Cl^−^ extrusion (Moore et al., 2018). Strikingly, KCC2-T906A/T1007A mice exhibited profound resistance to chemoconvulsant-induced seizures without altered basal neuronal excitability. These findings suggest that modulation of KCC2 phosphorylation sites may be leveraged to strengthen synaptic inhibition for therapeutic benefit in epilepsy syndromes. For clinical translation, KCC2 function could be enhanced by inhibition of the upstream with no lysine (WNK) and Ste20-related proline-alanine kinase (WNK-SPAK) cascade that normally inhibits KCC2 function by promoting T906 and T1007 phosphorylation (Kahle et al., 2005, 2014, 2016b; Friedel et al., 2015; Kahle and Delpire, 2016; Heubl et al., 2017). Indeed, small molecule inhibitors of the WNK-SPAK pathway are being developed (Yamada et al., 2016). In addition to targeting the WNK/SPAK pathway, other pharmacological strategies to enhance KCC2 activity are also being investigated. A large screening of molecules to identify putative KCC2 agonists was first proposed by Gagnon et al. (2013). Another screening was recently carried out to detect small molecules capable of enhancing KCC2 expression levels (Tang et al., 2019). The ability to augment KCC2 function *via* different pharmacological approaches will enable flexibility in the selection of the ideal KCC2 modulator that is tailored to the underlying epileptogenic process.

Although efforts to identify KCC2 modulators reflect its promise as a druggable target in epilepsy, there remain several caveats. First, it is unclear which pharmacological approach or combination of approaches to enhance KCC2 activity would rescue the defects in expression and function due to KCC2 mutations observed in epilepsy syndromes. Second, it remains uncertain whether there may be unintended adverse effects that arise from increased KCC2 activity. Indeed, a major shortcoming of current AEDs is not only their inability to prevent seizures in a large population of patients but also their association with drug-related side effects such as cognitive disturbance (Park and Kwon, 2008). Potentiating KCC2 activity does not alter basal neuronal excitability (Moore et al., 2018), and a previously identified KCC2 activator (CLP257) produces analgesia without motor side effects often seen with other analgesics (Gagnon et al., 2013). While these preliminary findings suggest that enhancing KCC2 may be a safe therapeutic avenue to prevent seizures without altering the function of healthy neurons, more systematic studies are still needed to fully characterize potential side effects of the approach before clinical translation.

## Conclusion

Epilepsy is a common brain disorder characterized by recurrent and unprovoked seizures thought to be caused by neuronal hyperexcitability. A third to half of epilepsy patients continue to have seizures despite medications (Kwan and Brodie, 2000; Shorvon and Luciano, 2007; Cascino, 2008), underscoring the clinical need for the identification of novel therapeutic targets. KCC2 functions as a major Cl^−^ extruder in mature neurons to establish an inwardly-directed electrochemical gradient of Cl^−^ necessary for the maintenance of fast synaptic inhibition. In the settings of diminished KCC2 activity secondary to risk factor and causal mutations in human epilepsy patients, intracellular Cl^−^ concentrations accumulate, leading to impaired hyperpolarizing responses that render neurons hyperexcitable. In contrast, increasing KCC2 function by overexpression or modulation of key phosphorylation sites confers an anticonvulsant effect. Altogether, the presence of KCC2 mutations in epilepsy coupled with preclinical proof-of-principle for KCC2 as a therapeutic target motivates a rich stream of future studies to further investigate the mechanistic roles of KCC2 in epileptogenesis and how manipulation of KCC2 activity can be leveraged pharmacologically for therapeutic benefit in epilepsy syndromes and conditions of hyperexcitation.

## Author Contributions

PD, WD and KK reviewed the literature and wrote the manuscript.

## Conflict of Interest

The authors declare that the research was conducted in the absence of any commercial or financial relationships that could be construed as a potential conflict of interest.

## References

[B1] Aldenkamp A. P. (2006). Cognitive impairment in epilepsy: state of affairs and clinical relevance. Seizure 15, 219–220. 10.1016/j.seizure.2006.02.010

[B2] Begley C. E. Famulari M. Annegers J. F. Lairson D. R. Reynolds T. F. Coan S.. (2000). The cost of epilepsy in the united states: an estimate from population-based clinical and survey data. Epilepsia 41, 342–351. 10.1111/j.1528-1157.2000.tb00166.x 10714408

[B3] Ben-Ari Y. Cherubini E. Corradetti R. Gaiarsa J. L. (1989). Giant synaptic potentials in immature rat CA3 hippocampal neurones. J. Physiol. 416, 303–325. 10.1113/jphysiol.1989.sp017762 2575165 PMC1189216

[B4] Cascino G. D. (2008). When drugs and surgery don’t work. Epilepsia 49, 79–84. 10.1111/j.1528-1167.2008.01930.x 19087121

[B5] Chen H.-Y. Albertson T. E. Olson K. R. (2016). Treatment of drug-induced seizures. Br. J. Clin. Pharmacol. 81, 412–419. 10.1111/bcp.12720 26174744 PMC4767205

[B6] Doyon N. Vinay L. Prescott S. A. De Koninck Y. (2016). Chloride regulation: a dynamic equilibrium crucial for synaptic inhibition. Neuron 89, 1157–1172. 10.1016/j.neuron.2016.02.030 26985723

[B7] Ellender T. J. Raimondo J. V. Irkle A. Lamsa K. P. Akerman C. J. (2014). excitatory effects of parvalbumin-expressing interneurons maintain hippocampal epileptiform activity via synchronous afterdischarges. J. Neurosci. 34, 15208–15222. 10.1523/JNEUROSCI.1747-14.2014 25392490 PMC4228130

[B8] Friedel P. Kahle K. T. Zhang J. Hertz N. Pisella L. I. Buhler E.. (2015). WNK1-regulated inhibitory phosphorylation of the KCC2 cotransporter maintains the depolarizing action of GABA in immature neurons. Sci. Signal. 8:ra65. 10.1126/scisignal.aaa0354 26126716

[B9] Gagnon M. Bergeron M. J. Lavertu G. Castonguay A. Tripathy S. Bonin R. P.. (2013). Chloride extrusion enhancers as novel therapeutics for neurological diseases. Nat. Med. 19, 1524–1528. 10.1038/nm.3356 24097188 PMC4005788

[B10] Hekmat-Scafe D. S. Lundy M. Y. Ranga R. Tanouye M. A. (2006). Mutations in the K^+^/Cl^−^ cotransporter gene *kazachoc (Kcc)* increase seizure susceptibility in *Drosophila*. J. Neurosci. 26, 8943–8954. 10.1523/JNEUROSCI.4998-05.2006 16943550 PMC6675325

[B11] Heubl M. Zhang J. Pressey J. C. Al Awabdh S. Renner M. Gomez-Castro F.. (2017). GABA_A_ receptor dependent synaptic inhibition rapidly tunes KCC2 activity via the Cl^−^-sensitive WNK1 kinase. Nat. Commun. 8:1776. 10.1038/s41467-017-01749-0 29176664 PMC5701213

[B12] Huberfeld G. Wittner L. Clemenceau S. Baulac M. Kaila K. Miles R.. (2007). Perturbed chloride homeostasis and GABAergic signaling in human temporal lobe epilepsy. J. Neurosci. 27, 9866–9873. 10.1523/JNEUROSCI.2761-07.2007 17855601 PMC6672644

[B13] Hübner C. A. Stein V. Hermans-Borgmeyer I. Meyer T. Ballanyi K. Jentsch T. J. (2001). Disruption of KCC2 reveals an essential role of K-Cl cotransport already in early synaptic inhibition. Neuron 30, 515–524. 10.1016/s0896-6273(01)00297-5 11395011

[B14] Imad H. Zelano J. Kumlien E. (2015). Hypoglycemia and risk of seizures: a retrospective cross-sectional study. Seizure 25, 147–149. 10.1016/j.seizure.2014.10.005 25455725

[B15] Kahle K. T. Delpire E. (2016). Kinase-KCC2 coupling: Cl^−^ rheostasis, disease susceptibility, therapeutic target. J. Neurophysiol. 115, 8–18. 10.1152/jn.00865.2015 26510764 PMC4760510

[B16] Kahle K. T. Khanna A. R. Duan J. Staley K. J. Delpire E. Poduri A. (2016a). The KCC2 cotransporter and human epilepsy: getting excited about inhibition. Neuroscientist 22, 555–562. 10.1177/1073858416645087 27130838

[B17] Kahle K. T. Schmouth J.-F. Lavastre V. Latremoliere A. Zhang J. Andrews N.. (2016b). Inhibition of the kinase WNK1/HSN2 ameliorates neuropathic pain by restoring GABA inhibition. Sci. Signal. 9:ra32. 10.1126/scisignal.aad0163 27025876 PMC5723157

[B19] Kahle K. T. Merner N. D. Friedel P. Silayeva L. Liang B. Khanna A.. (2014). Genetically encoded impairment of neuronal KCC2 cotransporter function in human idiopathic generalized epilepsy. EMBO Rep. 15, 766–774. 10.15252/embr.201438840 24928908 PMC4196980

[B18] Kahle K. T. Rinehart J. de Los Heros P. Louvi A. Meade P. Vazquez N.. (2005). WNK3 modulates transport of Cl^−^ in and out of cells: implications for control of cell volume and neuronal excitability. Proc. Natl. Acad. Sci. U S A 102, 16783–16788. 10.1073/pnas.0508307102 16275911 PMC1283843

[B21] Kaila K. Price T. J. Payne J. A. Puskarjov M. Voipio J. (2014). Cation-chloride cotransporters in neuronal development, plasticity and disease. Nat. Rev. Neurosci. 15, 637–654. 10.1038/nrn3819 25234263 PMC4294553

[B20] Kaila K. Voipio J. (1987). Postsynaptic fall in intracellular PH induced by GABA-activated bicarbonate conductance. Nature 330, 163–165. 10.1038/330163a0 3670401

[B22] Khirug S. Huttu K. Ludwig A. Smirnov S. Voipio J. Rivera C.. (2005). Distinct properties of functional KCC2 expression in immature mouse hippocampal neurons in culture and in acute slices. Eur. J. Neurosci. 21, 899–904. 10.1111/j.1460-9568.2005.03886.x 15787696

[B23] Kwan P. Brodie M. J. (2000). Early identification of refractory epilepsy. N. Engl. J. Med. 342, 314–319. 10.1056/NEJM200002033420503 10660394

[B24] Lee H. H. C. Deeb T. Z. Walker J. A. Davies P. A. Moss S. J. (2011). NMDA receptor activity downregulates KCC2 resulting in depolarizing GABA_A_ receptor-mediated currents. Nat. Neurosci. 14, 736–743. 10.1038/nn.2806 21532577 PMC3102766

[B25] Li H. Tornberg J. Kaila K. Airaksinen M. S. Rivera C. (2002). Patterns of cation-chloride cotransporter expression during embryonic rodent CNS development. Eur. J. Neurosci. 16, 2358–2370. 10.1046/j.1460-9568.2002.02419.x 12492431

[B26] Llano O. Smirnov S. Soni S. Golubtsov A. Guillemin I. Hotulainen P.. (2015). KCC2 regulates actin dynamics in dendritic spines via interaction with β-PIX. J. Cell Biol. 209, 671–686. 10.1083/jcb.201411008 26056138 PMC4460141

[B27] Magloire V. Cornford J. Lieb A. Kullmann D. M. Pavlov I. (2019). KCC2 overexpression prevents the paradoxical seizure-promoting action of somatic inhibition. Nat. Commun. 10:1225. 10.1038/s41467-019-08933-4 30874549 PMC6420604

[B28] Mefford H. C. Yendle S. C. Hsu C. Cook J. Geraghty E. McMahon J. M.. (2011). Rare copy number variants are an important cause of epileptic encephalopathies. Ann. Neurol. 70, 974–985. 10.1002/ana.22645 22190369 PMC3245646

[B29] Mercado A. Broumand V. Zandi-Nejad K. Enck A. H. Mount D. B. (2006). A C-terminal domain in KCC2 confers constitutive K^+^-Cl^−^ cotransport. J. Biol. Chem. 281, 1016–1026. 10.1074/jbc.M509972200 16291749

[B30] Moore E. Y. Deeb T. Z. Chadchankar H. Brandon N. J. Moss S. J. (2018). Potentiating KCC2 activity is sufficient to limit the onset and severity of seizures. Proc. Natl. Acad. Sci. U S A 115, 10166–10171. 10.1073/pnas.1810134115 30224498 PMC6176565

[B31] Moore Y. E. Kelley M. R. Brandon N. J. Deeb T. Z. Moss S. J. (2017). Seizing control of KCC2: a new therapeutic target for epilepsy. Trends Neurosci. 40, 555–571. 10.1016/j.tins.2017.06.008 28803659

[B32] Park S.-P. Kwon S.-H. (2008). Cognitive effects of antiepileptic drugs. J. Clin. Neurol. 4, 99–106. 10.3988/jcn.2008.4.3.99 19513311 PMC2686875

[B33] Payne J. A. Rivera C. Voipio J. Kaila K. (2003). Cation-chloride co-transporters in neuronal communication, development and trauma. Trends Neurosci. 26, 199–206. 10.1016/s0166-2236(03)00068-7 12689771

[B34] Pershad D. Siddiqui R. S. (1992). Psychosocial dysfunction in adults with epilepsy. Int. J. Rehabil. Res. 15, 258–261. 10.1097/00004356-199209000-00012 1428394

[B35] Perucca E. Meador K. J. (2005). Adverse effects of antiepileptic drugs. Acta Neurol. Scand. Suppl. 181, 30–35. 10.1111/j.1600-0404.2005.00506.x 16238706

[B36] Plotkin M. D. Snyder E. Y. Hebert S. C. Delpire E. (1997). Expression of the Na-K-2Cl cotransporter is developmentally regulated in postnatal rat brains: a possible mechanism underlying GABA’s excitatory role in immature brain. J. Neurobiol. 33, 781–795. 10.1002/(sici)1097-4695(19971120)33:6<781::aid-neu6>3.0.co;2-5 9369151

[B37] Puskarjov M. Seja P. Heron S. E. Williams T. C. Ahmad F. Iona X.. (2014). A variant of KCC2 from patients with febrile seizures impairs neuronal Cl^−^ extrusion and dendritic spine formation. EMBO Rep. 15, 723–729. 10.1002/embr.201438749 24668262 PMC4197883

[B38] Rinehart J. Maksimova Y. D. Tanis J. E. Stone K. L. Hodson C. A. Zhang J.. (2009). Sites of regulated phosphorylation that control K-Cl cotransporter activity. Cell 138, 525–536. 10.1016/j.cell.2009.05.031 19665974 PMC2811214

[B39] Saito T. Ishii A. Sugai K. Sasaki M. Hirose S. (2017). A *de novo* missense mutation in SLC12A5 found in a compound heterozygote patient with epilepsy of infancy with migrating focal seizures. Clin. Genet. 92, 654–658. 10.1111/cge.13049 28477354

[B40] Saitsu H. Watanabe M. Akita T. Ohba C. Sugai K. Ong W. P.. (2016). Impaired neuronal KCC2 function by biallelic SLC12A5 mutations in migrating focal seizures and severe developmental delay. Sci. Rep. 6:30072. 10.1038/srep30072 27436767 PMC4951812

[B41] Sander J. W. Shorvon S. D. (1996). Epidemiology of the epilepsies. J. Neurol. Neurosurg. Psychiatry 61, 433–443. 10.1136/jnnp.61.5.433 8965090 PMC1074036

[B42] Sedmak G. Jovanov-Miloševic N. Puskarjov M. Ulamec M. Krušlin B. Kaila K.. (2016). Developmental expression patterns of KCC2 and functionally associated molecules in the human brain. Cereb. Cortex 26, 4574–4589. 10.1093/cercor/bhv218 26428952

[B43] Shorvon S. Luciano A. L. (2007). Prognosis of chronic and newly diagnosed epilepsy: revisiting temporal aspects. Curr. Opin. Neurol. 20, 208–212. 10.1097/wco.0b013e3280555175 17351493

[B44] Stafstrom C. E. Carmant L. (2015). Seizures and epilepsy: an overview for neuroscientists. Cold Spring Harb. Perspect. Med. 5:a022426. 10.1101/cshperspect.a022426 26033084 PMC4448698

[B45] Stein V. Hermans-Borgmeyer I. Jentsch T. J. Hübner C. A. (2004). Expression of the KCl cotransporter KCC2 parallels neuronal maturation and the emergence of low intracellular chloride. J. Comp. Neurol. 468, 57–64. 10.1002/cne.10983 14648690

[B46] Stödberg T. McTague A. Ruiz A. J. Hirata H. Zhen J. Long P.. (2015). Mutations in SLC12A5 in epilepsy of infancy with migrating focal seizures. Nat. Commun. 6:8038. 10.1038/ncomms9038 26333769 PMC4569694

[B47] Tang X. Drotar J. Li K. Clairmont C. D. Brumm A. S. Sullins A. J.. (2019). Pharmacological enhancement of KCC2 gene expression exerts therapeutic effects on human rett syndrome neurons and Mecp2 mutant mice. Sci. Transl. Med. 11:eaau0164. 10.1126/scitranslmed.aau0164 31366578 PMC8140401

[B48] Tanis J. E. Bellemer A. Moresco J. J. Forbush B. Koelle M. R. (2009). The potassium chloride cotransporter KCC-2 coordinates development of inhibitory neurotransmission and synapse structure in caenorhabditis elegans. J. Neurosci. 29, 9943–9954. 10.1523/JNEUROSCI.1989-09.2009 19675228 PMC2737711

[B49] Till Á. Szalai R. Hegyi M. Kövesdi E. Büki G. Hadzsiev K.. (2019). A rare form of ion channel gene mutation identified as underlying cause of generalized epilepsy. Orv. Hetil. 160, 835–838. 10.1556/650.2019.31404 31104500

[B50] Titz S. Sammler E. M. Hormuzdi S. G. (2015). Could tuning of the inhibitory tone involve graded changes in neuronal chloride transport? Neuropharmacology 95, 321–331. 10.1016/j.neuropharm.2015.03.026 25843644

[B51] Williams J. R. Sharp J. W. Kumari V. G. Wilson M. Payne J. A. (1999). The neuron-specific K-Cl cotransporter, KCC2. Antibody development and initial characterization of the protein. J. Biol. Chem. 274, 12656–12664. 10.1074/jbc.274.18.12656 10212246

[B400] World Health Organization (2019). “Neurological disorders, including epilepsy,” in Mental Health Home. Available online at: https://www.who.int/mental_health/management/neurological/en/.

[B52] Yamada J. Okabe A. Toyoda H. Kilb W. Luhmann H. J. Fukuda A. (2004). Cl^−^ uptake promoting depolarizing GABA actions in immature rat neocortical neurones is mediated by NKCC1. J. Physiol. 557, 829–841. 10.1113/jphysiol.2004.062471 15090604 PMC1665166

[B5200] Yamada K. Zhang J.-H. Xie X. Reinhardt J. Xie A. Q. LaSala D.. (2016). Discovery and characterization of allosteric WNK kinase inhibitors. ACS Chem. Biol. 11, 3338–3346. 10.1021/acschembio.6b00511 27712055

[B53] Zieliński J. J. (1974). Epilepsy and mortality rate and cause of death. Epilepsia 15, 191–201. 10.1111/j.1528-1157.1974.tb04941.x 4525178

[B54] Zoons E. Weisfelt M. de Gans J. Spanjaard L. Koelman J. H. T. M. Reitsma J. B.. (2008). Seizures in adults with bacterial meningitis. Neurology 70, 2109–2115. 10.1212/01.wnl.0000288178.91614.5d 18305232

